# Does the “surprisingly popular” method yield accurate crowdsourced predictions?

**DOI:** 10.1186/s41235-020-00256-z

**Published:** 2020-11-11

**Authors:** Abraham M. Rutchick, Bryan J. Ross, Dustin P. Calvillo, Catherine C. Mesick

**Affiliations:** 1 California State University, Northridge, Northridge, USA; 2 California State University San Marcos, San Marcos, USA

**Keywords:** Surprisingly popular method, Wisdom of crowds, Prediction, Forecasting, Crowdsourcing

## Abstract

The “surprisingly popular” method (SP) of aggregating individual judgments has shown promise in overcoming a weakness of other crowdsourcing methods—situations in which the majority is incorrect. This method relies on participants’ estimates of other participants’ judgments; when an option is chosen more often than the average metacognitive judgments of that option, it is “surprisingly popular” and is selected by the method. Although SP has been shown to improve group decision making about factual propositions (e.g., state capitals), its application to future outcomes has been limited. In three preregistered studies, we compared SP to other methods of aggregating individual predictions about future events. Study 1 examined predictions of football games, Study 2 examined predictions of the 2018 US midterm elections, and Study 3 examined predictions of basketball games. When applied to judgments made by objectively assessed experts, SP performed slightly better than other aggregation methods. Although there is still more to learn about the conditions under which SP is effective, it shows promise as a means of crowdsourcing predictions of future outcomes.

## Significance statement

When judgments are combined, the result is often more accurate than the individual judgments by themselves—the “wisdom of the crowd” phenomenon. For example, the average estimate (in a classic case, of the weight of an ox) is more accurate than most individual estimates, and the Las Vegas point spread, which is driven by gamblers’ collective decisions, often accurately predicts the winners of games. Sometimes, however, the majority is wrong. For example, most people erroneously believe that Los Angeles, California, is west of Reno, Nevada, and most people incorrectly predicted the 2016 US Presidential election. A recently developed approach, the “surprisingly popular” method, constructs group predictions such that minority opinions can influence the collective choice. When people are correct but in the minority, they often know that many others do not know the correct answer. This knowledge can be leveraged by asking people to make one additional judgment: the percentage of other participants who will make the same judgment they did. Integrating this judgment into the group decision allows the minority choice to sometimes be selected. However, this method has usually been applied to factual judgments, such as knowledge of state capitals, and only rarely has examined prediction of future events. We applied this method to predictions of football games, US elections, and basketball games. We found that the “surprisingly popular” method indeed yielded the most accurate collective predictions, but only when the people making the predictions were knowledgeable about the subject.

## Introduction

Aggregations of judgments often outperform those of individuals. This phenomenon, often termed “the wisdom of crowds” (Surowiecki 2005), has been shown in many decision and prediction contexts, including mathematical problems (Yi et al. 2012), game shows (Lee et al. 2011), and elections (Gaissmaier and Marewski 2011). However, most crowdsourcing methods have an important limitation—they cannot detect cases in which the majority is wrong.

One recently developed aggregation approach, the “surprisingly popular” method (Prelec et al. 2017; henceforth “SP”), has shown promise in overcoming this weakness. The SP method leverages metacognitive awareness: people who are correct, but in the minority, often know that their response is rare. Participants answer one additional question: the percentage of other participants who will make the same judgment they did. These estimates are then compared to participants’ actual judgments. When an option is chosen more often than the average metacognitive judgments of that option, it is “surprisingly popular” and is selected by the method.

For example, suppose participants are asked whether Reno, Nevada, is east of Los Angeles, California. Because most of Nevada is east of most of California, people often respond that Reno is east of Los Angeles. This is incorrect; Reno is some 86 miles west of Los Angeles. Suppose that 30% of people know this. They also—importantly—know that this knowledge is rare, and estimate, on average, that 15% of others are also correct. Now consider the 70% of people who are incorrect; suppose that they believe, on average, that 90% of others agree with their answer. Thus, although the average metajudgment was that only 11.5%^1^ of people believe that Reno is west of Los Angeles, that answer was actually given by 30% of respondents, making it “surprisingly popular.”

Most demonstrations of the SP method have examined judgments in which the correct answer is known. Although improving the accuracy of such judgments may inform understanding of judgments about as-yet-unsolved questions, it does not necessarily follow that improvements in problem solving imply improvements in prediction. Leveraging the SP method to improve prediction of future events is a particularly exciting potential application of this approach.

Lee et al. (2018) provided the first test of whether the SP method can improve collective judgments of *unknown* events—that is, future outcomes. Lee et al. (2018) had participants predict the winners of National Football League (NFL) games in the 2017 season. They found that, among participants who indicated that they were “extremely knowledgeable” about football, the SP method yielded better predictions than many NFL media figures, an alternative aggregative method (confidence-weighted judgments), and a prominent algorithmic approach to prediction (by fivethirtyeight.com). However, SP was inferior to the democratic method (the modal judgment). Given these mixed results, Lee et al. (2018) were appropriately cautious in their conclusions. First, they noted that participants were capable of easily making metacognitive judgments about future events, as they are in the case of factual judgments. Second, they emphasized the importance of expertise in yielding accurate predictions using the SP method. However, several important questions remain unanswered.

First, does the SP method actually yield more accurate predictions than other aggregation methods? Examining Lee et al. (2018), the most straightforward implication is that SP does not clearly outperform other approaches. Nevertheless, it may be that the particular NFL season examined by Lee et al. (2018) is not representative of future events, sporting events, or even NFL seasons. Thus, it remains useful to provide additional tests of the SP method.

Second, does the SP method perform better when it aggregates judgments made by experts? Prelec et al. (2017) did not find systematic differences in the effectiveness of the SP method based on expertise. In contrast, Lee et al. (2018) found that the SP method was more effective when it aggregated only judgments made by self-assessed experts. However, this selection decision was exploratory (Lee et al. 2018, p. 326), and moreover, self-assessments of expertise are not always accurate (Kruger and Dunning 1999).

To examine these questions, we conducted three studies in which participants predicted future outcomes. We compared the SP method to other methods of aggregating crowdsourced judgments and also assessed expertise by testing domain knowledge. Study 1 examined predictions of NFL games made by students, Study 2 examined predictions of the 2018 midterm elections made by mTurk workers, and Study 3 examined predictions of NBA games made by members of the /r/NBA and /r/sportsbook subreddits and students in a sport psychology course. We hypothesized that the SP method, when applied to judgments made by experts, would yield more accurate forecasts than other crowdsourcing approaches.

## Study 1

In Study 1, we replicated Lee et al. (2018), with two important refinements. First, we preregistered our procedure for selecting experts and our decision to analyze experts separately (https://osf.io/u9k72/; preregistrations, materials, and data for all studies can be found there). Second, we included an objective method of assessing expertise.

## Method

### Participants

Participants were recruited from a psychology course and were compensated with extra credit. All participants (*N* = 227) completed a survey at the outset of the NFL season; 205 made at least one prediction. The maximum number of participants in a week was 161; the minimum was 121.

### Materials and procedure

All participants completed a survey with demographics, a self-evaluation of NFL knowledge on a 5-point scale (from “not knowledgeable at all,” to “extremely knowledgeable,” per Lee et al. 2018), and a 31-question NFL knowledge questionnaire (based on Van Overschelde et al. 2005); the questionnaire is available on the OSF page.

Each Tuesday during the NFL season, participants received a survey presenting that week’s NFL games in chronological order. Participants predicted the winner of each game, indicated their confidence in that prediction on a 1 (guess) to 5 (very high confidence) scale, and estimated the percentage of other participants who agreed with their prediction.

### Differences from Lee et al. (2018)

As noted above, Study 1 included an objective measure of expertise. Study 1 also differed from Lee et al. (2018) in two other ways. First, it used a student sample rather than mTurk workers. Second, it examined only part of the 2018 NFL season (weeks 1 through 15) due to the end of the semester during which student participants were available, rather than the entire 17-week 2017 NFL season. For eight games, the incorrect team was listed as the home team; these were excluded, yielding 216 which were analyzed.

## Results and discussion

### Aggregation approaches

Predictions were aggregated using three methods (following Lee et al. 2018). First, the *democratic* method selects the team who was predicted to win by the most participants. Continuing the example discussed previously, because 70% of people believed that Reno is east of Los Angeles, the democratic method would select this (incorrect) response. Second, the *confidence-weighted* method multiplied each prediction by its associated confidence and selected the team with the highest weighted prediction count. Suppose that, in the Reno/Los Angeles example, the 30% of people correctly responding that Reno was west of Los Angeles had a mean confidence of 4.5 in their choice, whereas the remaining 70% had a mean confidence of 3.5. Here, the confidence-weighted method would select the incorrect response.^2^ Last, the *surprisingly* *popular* (SP) method, described previously, identified the option chosen more often by participants than it was estimated to be chosen. When a method resulted in a tie, 0.5 correct predictions were awarded.

In addition to the aggregation approaches, the predictions made by members of the media (as recorded by nflpickwatch.com) were recorded. The predictions made by fivethirtyeight.com, which were generated algorithmically, were recorded as an additional point of comparison.

### Whole-sample analyses

**Fig. 1:**
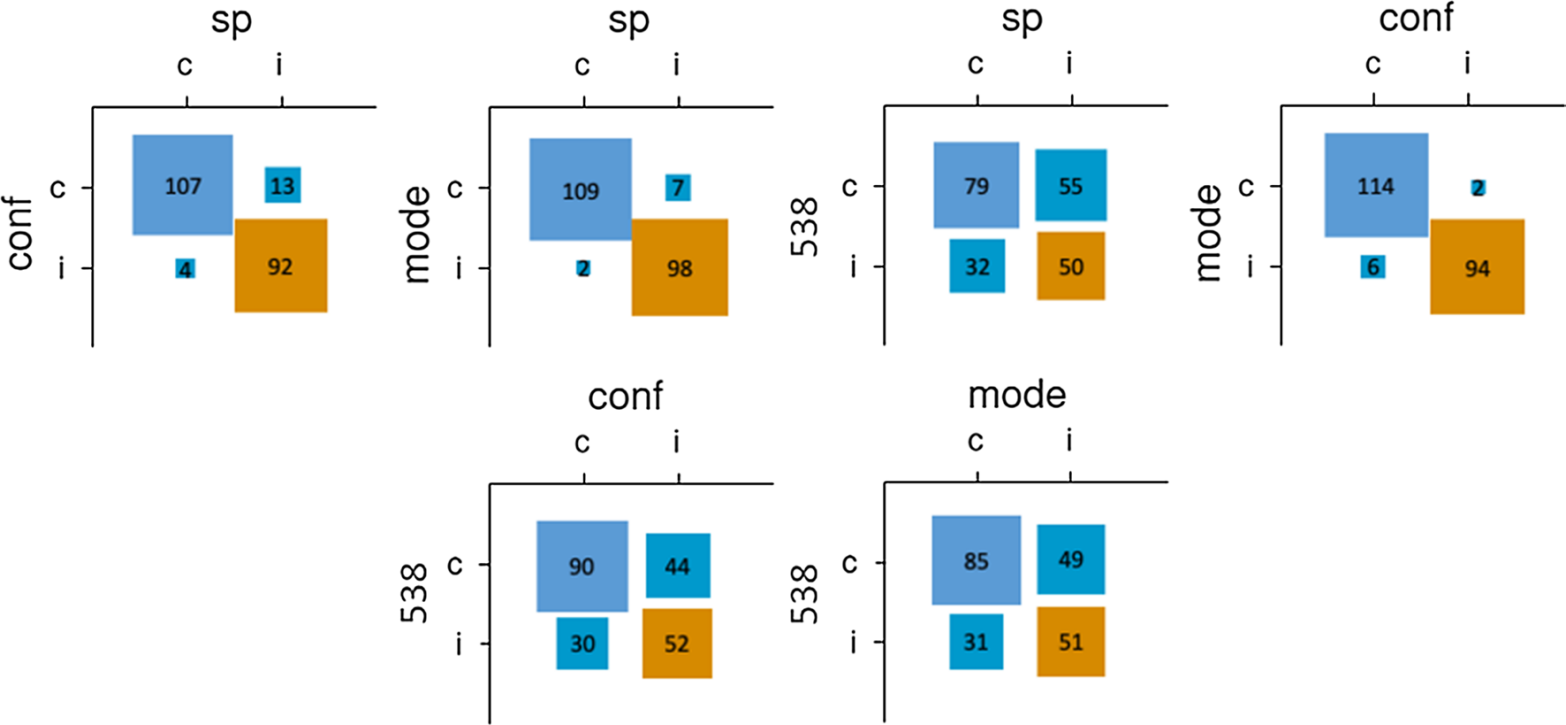
Relationships between pairs of prediction methods for Study 1 (total sample). *Note* sp = surprisingly popular; conf = confidence-weighted; mode = democratic; 538 = Fivethirtyeight.com. Correct predictions are labeled as “c,” and incorrect predictions are labeled as “i,” with the top-left square indicating correct predictions from both methods, the bottom-right square indicating incorrect predictions from both methods, the bottom-left square indicating correct predictions from the left-labeled method but not the top-labeled method, and the top-right square indicating correct predictions from the top-labeled method but not the left-labeled method

Individual participants’ predictions were correct 52.8% of the time. The democratic method outperformed individual predictions only narrowly (53.9% correct; Table 1); the confidence-weighted method performed better (55.6%), and the SP method performed worse (51.4%). As shown in Fig. 1, the three aggregative methods made quite similar predictions, deviating little from one another. Conversely, the algorithmically generated predictions made by fivethirtyeight.com differed strikingly from the crowdsourced methods and were more accurate (62.0%).

**Table 1:** Performance (correct predictions, % correct) across aggregation methods

	Individual	Democratic	Confidence-weighted	Surprisingly popular
Total sample	52.8%	116.5, 53.9%	120, 55.6%	111, 51.4%
Objectively assessed experts	54.9%	127.5, 59%	129, 59.7%	130, 60.2%
NFLpickwatch.com	62.3%	140.5, 65.0%		
Fivethirtyeight.com	134, 62.0%

### Self-assessed experts

Following the approach used by Lee et al. (2018), which we preregistered, participants who rated their knowledge of NFL football as “extremely knowledgeable” were considered *self-assessed* *experts.* There were only 6 such participants (2.6% of the sample); the number who made predictions ranged from 3 to 5. Because there were too few self-assessed experts to produce reliable crowdsourced predictions, we report results for this sample in Additional file 1.

### Objectively assessed experts

**Fig. 2:**
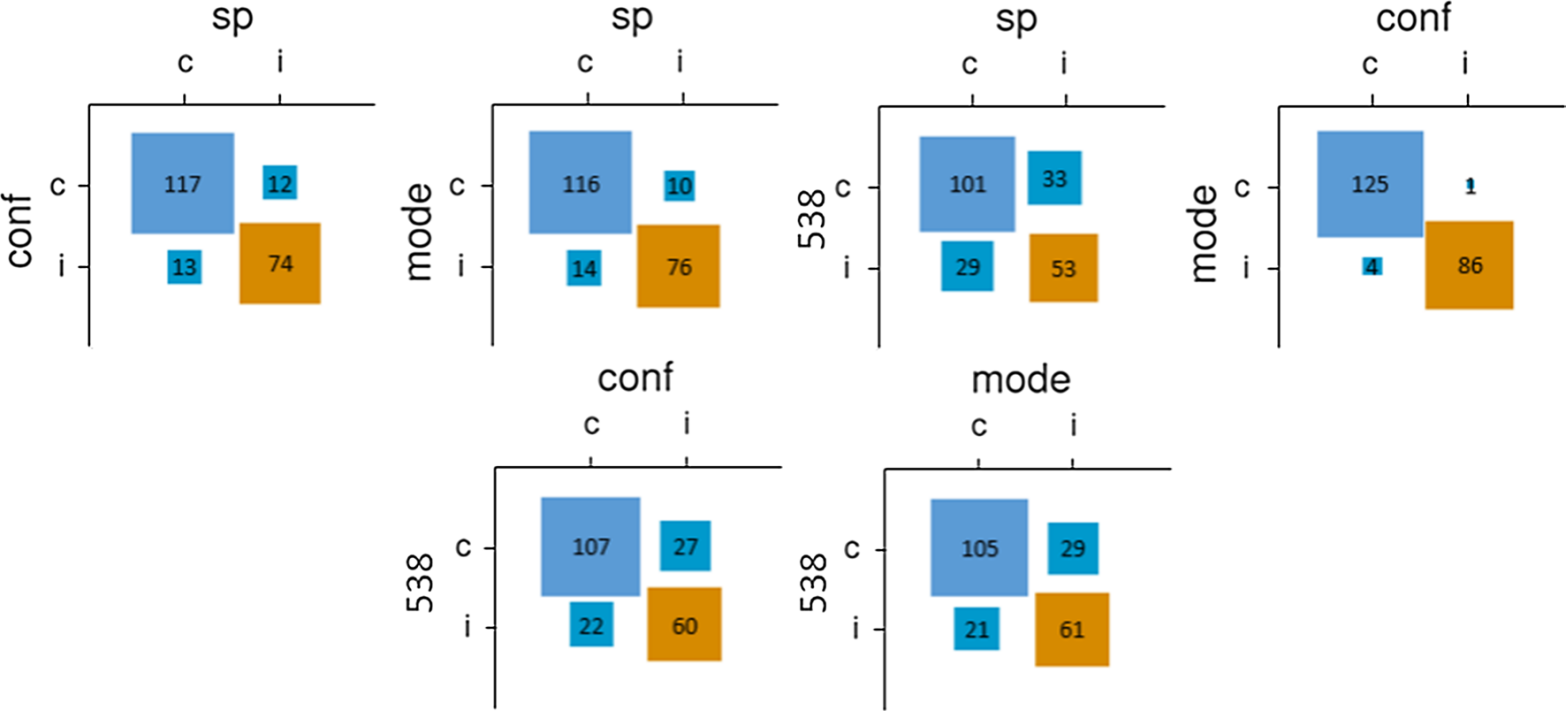
Relationships between pairs of prediction methods for Study 1 (objectively assessed experts). *Note* sp = surprisingly popular; conf = confidence-weighted; mode = democratic; 538 = fivethirtyeight.com

Per the preregistration, participants scoring in the top quintile of the knowledge quiz were considered objectively assessed experts; 46 participants scored above the 80th percentile (22/30), with the number making predictions each week ranging from 27 to 37. Aggregations of objectively assessed experts outperformed the overall sample (and the subsample of self-assessed experts) regardless of the method used. This improvement in performance ranged from 9 games (4.2%) for the confidence-weighted method to 19 games (8.8%) for the SP method. Comparing across aggregative methods, the SP method performed best, although only narrowly. As shown in Fig. 2, the SP method made predictions that were relatively distinct from those made by the other two crowdsourced methods (which were quite similar to one another). As was the case in the whole sample, fivethirtyeight.com’s algorithmic predictions differed sharply from all three crowdsourced methods. Although the SP method was the most successful crowdsourced method, it was also the most distinct from the algorithmic method.

In sum, the SP method did not perform well when used to aggregate the judgments made by the total sample. When applied to an objectively assessed expert subsample, SP was the best-performing method. All of these methods were outperformed by the predictions of media members covering the NFL, the democratic aggregation of those predictions, and the modeling-based approach of fivethirtyeight.com.

## Study 2

Study 2 examined the SP method in another domain: US elections. As in Study 1, we conducted parallel analyses on subjectively and objectively defined expert subsamples. The study was preregistered.

## Method

### Participants

Participants were recruited from Amazon’s Mechanical Turk; a total of 401 participants were recruited and compensated $1. Ninety-two participants failed preregistered quality control checks and were excluded, leaving 309 participants whose data were analyzed.

### Materials and procedure

We selected 41 of the 435 Congressional races in the 2018 US midterm elections, endeavoring to choose races that were high-profile and competitive, as well as the 33 Senate races and 36 gubernatorial races; this yielded 110 total races. Each participant predicted 25 randomly selected races, which yielded from 63 to 76 predictions per race. Predictions were made on November 1, 2018, five days before the election. Participants first reported demographic information and rated their knowledge of politics, then completed a political knowledge questionnaire consisting of 14 questions, which was an updated version of that used by Miller et al. (2016). Participants then made the same judgments as in Study 1 (prediction, confidence, agreement) about each race.

## Results and discussion

### Total-sample analyses

**Fig. 3:**
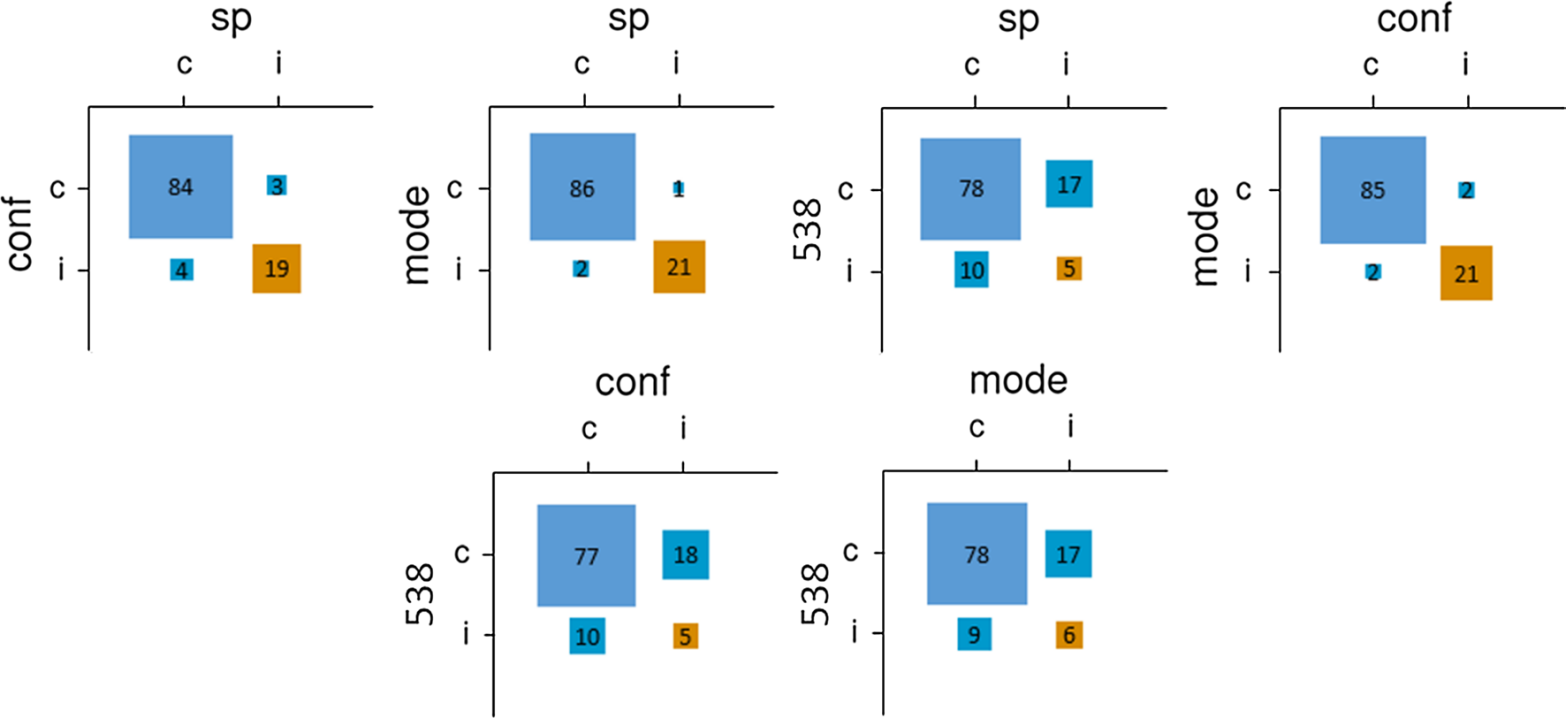
Relationships between pairs of prediction methods for Study 2 (total sample). *Note* sp = surprisingly popular; conf = confidence-weighted; mode = democratic; 538 = fivethirtyeight.com

Individual predictions were correct 60.8% of the time. When examining the total sample, the democratic method performed quite well (87/110 races predicted correctly or 79.1%; see Table 2). The confidence-weighted and SP methods each improved predictions slightly over the democratic method (we preregistered inferential tests comparing the methods’ accuracy; given the extremely low sensitivity of these tests, we now discuss them in Additional file 1). This finding is somewhat consistent with Study 1, in that all three methods performed similarly, although here all aggregative methods performed much better than individual forecasts. As shown in Fig. 3, there was considerable overlap among the methods’ predictions, with fivethirtyeight.com again diverging notably from the crowdsourced models. All methods performed worse than the weighted integration of polling published by fivethirtyeight.com, which predicted 95/110 races (86.4%) correctly.

**Table 2:** Performance (correct predictions, % correct) across aggregation methods

	Individual	Democratic	Confidence-weighted	Surprisingly popular
Total sample	60.8%	87, 79.1%	87.5, 79.6%	88, 80.0%
Self-assessed experts	68.2%	85, 77.3%	84, 76.4%	79, 71.8%
Objectively assessed experts	67.0%	87.5, 79.6%	88, 80.0%	91, 82.7%
Fivethirtyeight.com	95, 86.4%

### Self-assessed experts

**Fig. 4:**
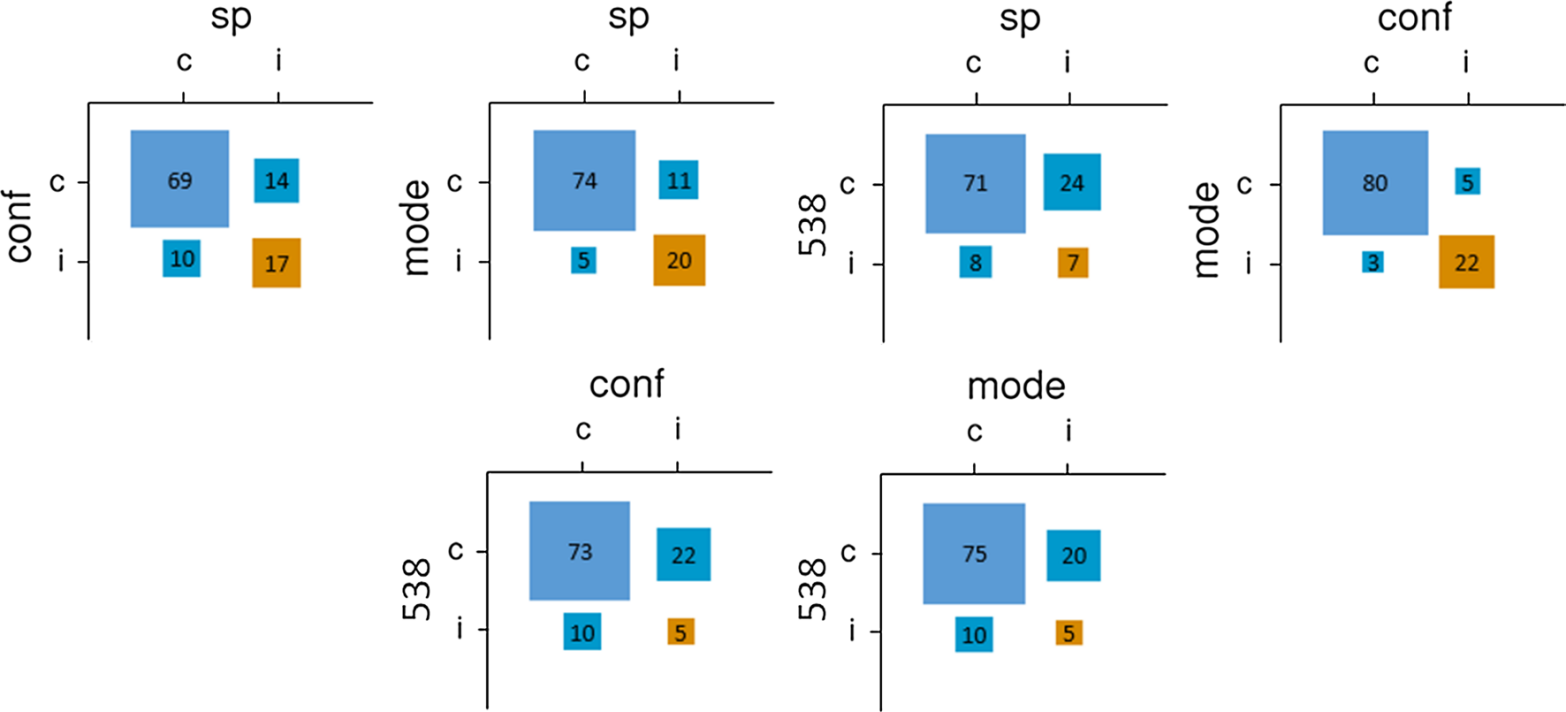
Relationships between pairs of prediction methods for Study 2 (self-assessed experts). *Note* sp = surprisingly popular; conf = confidence-weighted; mode = democratic; 538 = fivethirtyeight.com

As in Study 1, participants who indicated that they were “extremely knowledgeable” were considered self-assessed experts. Thirty-two participants (10.4% of the sample) satisfied this criterion. Aggregating the judgments of self-assessed experts yielded worse predictions than aggregating the overall sample. Comparing across methods, the confidence-weighted method did not improve predictions over the democratic method, and the SP method performed worse than the other methods. Figure 4 depicts the pairwise accuracies and prediction overlap among methods; consistent with the whole-sample predictions, the crowdsourced methods’ predictions overlapped considerably and fivethirtyeight.com’s predictions deviated notably from them.

### Objectively assessed experts

**Fig. 5:**
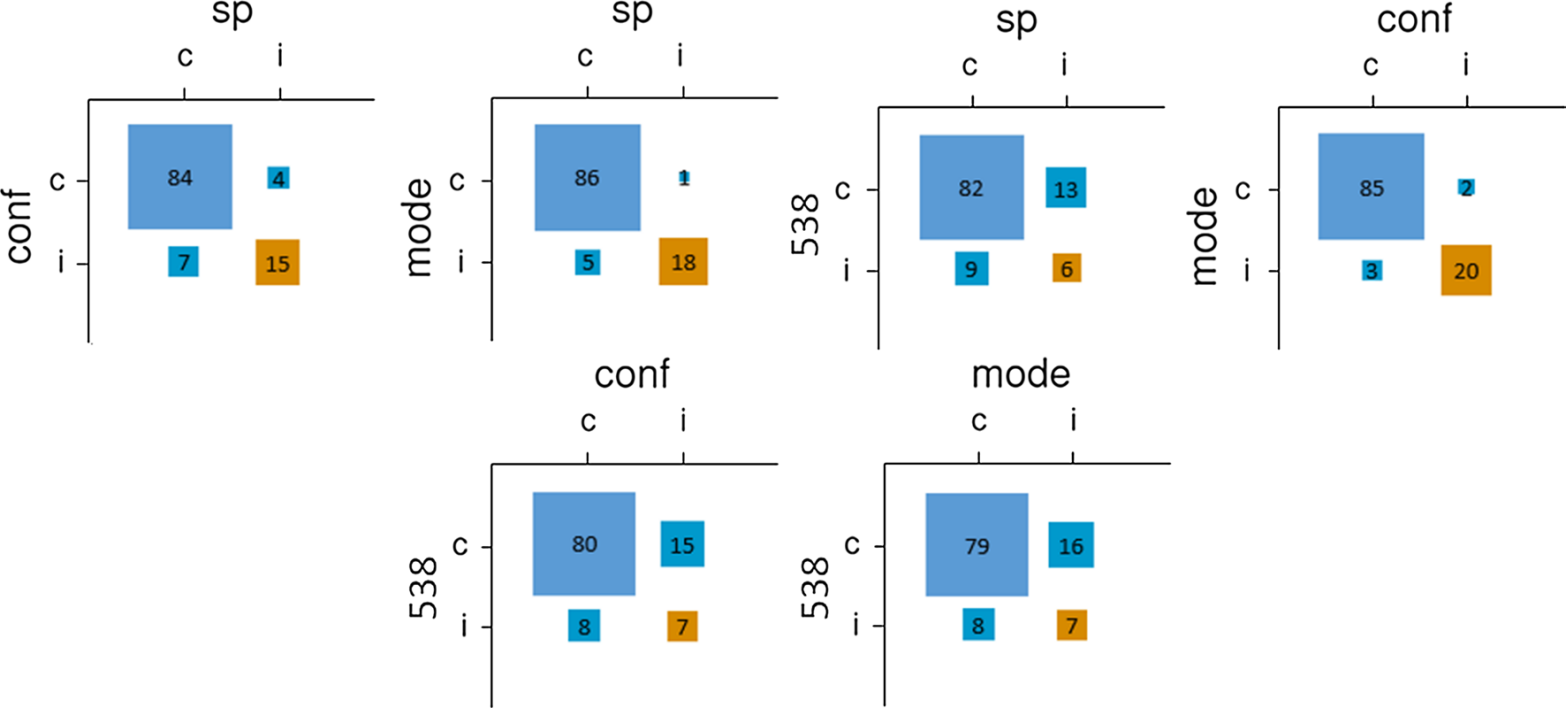
Relationships between pairs of prediction methods for Study 2 (objectively assessed experts). *Note* sp = surprisingly popular; conf = confidence-weighted; mode = democratic; 538 = fivethirtyeight.com

Per the preregistration, participants in the top quintile of the knowledge questionnaire were to be considered objectively assessed experts. However, 116 participants received a perfect score of 14/14, and thus the 62.5^th^ percentile was used as the criterion for objective expertise. In contrast to Study 1, the democratic and confidence-weighted methods yielded the same accuracy as in the full sample. However, the SP method did yield ordinally more accurate predictions and was the best-performing method within this subsample. In Fig. 5, consistent with the other subsamples in Study 2, there was considerable overlap among crowdsourced predictions, and fivethirtyeight.com’s predictions were notably different from them.

## Study 3

Study 3 was a conceptual replication of Study 1, examining predictions of basketball games rather than football games. Participants in Study 3 were sampled from populations that were considerably more expert than those in Study 1.

## Method

### Participants

We recruited participants by posting on the /r/NBA and /r/sportsbook subreddits, discussion boards for professional basketball and gambling on sports contests. These participants were supplemented by recruiting from a course on sport psychology. All participants (*N* = 130) completed an initial survey at the outset of the study; 111 made at least one prediction. Each week, the participant who accurately predicted the most game results and the participant who most accurately judged other participants’ predictions were each rewarded with a $10 Amazon.com gift card.

### Materials and Procedure

As in Studies 1 and 2, participants first completed a basketball knowledge assessment. This 19-item questionnaire (available on OSF) examined knowledge of the rules and history of NBA basketball. Then, beginning on January 20th (the 14th week of the 2018–2019 NBA season^3^), participants predicted games. After the first week, participant feedback suggested that predicting the full week’s slate was too time-consuming. Thus, after January 27th, surveys were sent twice per week, and participants predicted half a week at a time.

We preregistered the decision to exclude games in which the number of participants was less than 25% of the peak number (as games with very few predictions yield noisier results, and the analyses weight all games equally). Following this criterion, half of weeks 6, 11, and 12 were excluded. The remaining sample consisted of 465 games.

### Expertise of the sample

Study 3 participants had, on average, 12.6 correct answers (SD = 3.86) on the 19-item questionnaire. To assess whether this score implied expertise, the questionnaire was administered to a sample drawn from the same course used in Study 1 and to a mTurk sample. Both students (*N* = 207, *M* = 3.39, SD = 3.45, *t*(324) = 22.78, *p* < 0.01, Cohen’s *d* = 2.52) and mTurk workers (*N* = 532, *M* = 3.73, SD = 3.89, *t*(660) = 23.34, *p* < 0.01, Cohen’s *d* = 2.29) performed strikingly worse than Study 3 participants, suggesting that participants in Study 3 were quite knowledgeable about basketball (or, at minimum, much more so than the participants in Studies 1 and 2).

## Results and discussion

Although we preregistered an analytic plan to examine expert subsamples in the same way in which the full sample was examined, we now present these analyses in Additional file 1 rather than in the main text. Of the 130 total participants, 21 rated themselves as “extremely knowledgeable,” and 18 of these made predictions. This subsample yielded from 1 to 9 predictions each week, which was too small to reliably examine. There were 38 objectively assessed experts (at least 15/19 correct answers); 34 made predictions, which yielded 2 to 22 predictions each week. Thus, we focused on the overall sample, which was already composed of basketball experts.

**Figure 6:**
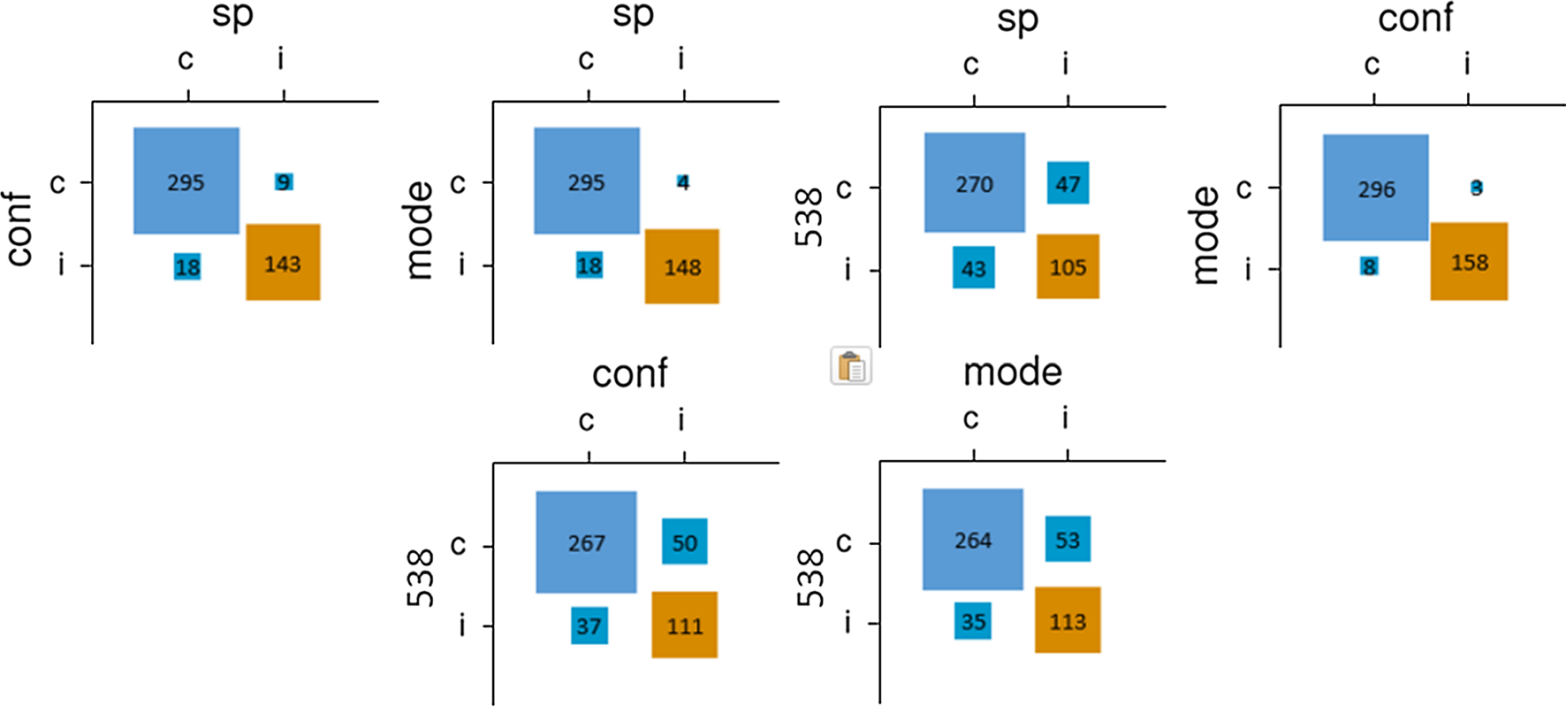
Relationships between pairs of prediction methods for Study 3 (total sample). *Note* sp = surprisingly popular; conf = confidence-weighted; mode = democratic; 538 = fivethirtyeight.com.

Individual predictions were correct 61.8% of the time. The democratic (306.5, 65.9%) and confidence-weighted methods (304.5 correct predictions, 65.5%) both improved slightly on individual predictions. They were outperformed by the SP method (313 correct predictions, or 67.3%) and fivethirtyeight.com (317, 68.2%). As shown in Fig. 6, the SP method’s predictions differed more from those of the other two crowdsourced methods than they did from each other. As in previous studies, fivethirtyeight.com’s algorithmic predictions differed sharply from all three crowdsourced methods. Although the SP method was the most accurate crowdsourced method, it was also the most distinct from the algorithmic method (albeit narrowly), a pattern that echoes that observed among objective experts in Study 1.

## General discussion

In three studies, we examined various ways to aggregate individual predictions into collective judgments of future events, focusing on the recently developed “surprisingly popular” method. Specifically, we investigated both the method’s overall effectiveness and whether it was more effective when used with expert samples. Our findings were inconclusive, although the SP method did yield the most accurate predictions when applied to the judgments of experts.

When applied to samples without specialized knowledge, there was no evidence that the SP method produced more accurate predictions than other aggregation methods, consistent with Lee et al. (2018). Although the SP method has been effectively applied to aggregate the judgments of non-experts (Prelec et al. 2017), the judgments in those studies applied to questions with known answers, such as state capitals. It may be that expertise matters more in instances of “true” prediction of as-yet-unknown outcomes.

Because the SP method relies on metacognition—participants’ ability to both make predictions and know whether those predictions are shared—it should be particularly effective when used to aggregate the judgments of experts, whose knowledge and, presumably, domain-specific metaknowledge is superior (MacIntyre et al. 2014). This hypothesis was supported. In Study 1, the SP method was most effective when applied to objectively assessed experts. In Study 2, using the SP method to aggregate the predictions of objectively assessed experts yielded the most accurate predictions of any crowdsourced approach. In Study 3, which examined a sample of experts, the SP method also yielded the most accurate predictions. Generally, then, it seems that—at least when expertise is assessed objectively—the SP method was more effective when applied to expert participants. This is consistent with extant work examining aggregation of expert judgments (c.f. Mannes et al. 2014). However, it should be noted that only in Studies 2 and 3 did aggregative methods (in general) decisively outperform individual judgments.

Although the SP method performed ordinally better than other methods when aggregating expert judgments, the differences between methods were small. However, small differences in predictive accuracy can still be consequential. To concretize this, consider the 2019 Westgate SuperContest, in which contestants paid a $10,000 entry fee and picked five NFL games each week against the spread. The average accuracy of the participants who predicted all games was 50.4%. The top 100 of the 3123 entrants won cash prizes; the average accuracy of these entrants was 61.7%. Or, considering another metric, suppose $100 was wagered on each of the contests in all three studies, using each method; assuming a − 110 vigorish on all bets, betting on the outcome chosen by the surprisingly popular aggregation of objectively assessed experts would have yielded $2300 more profit than the next-highest method.

The current studies had several limitations. First, participants in Studies 1 and 2 may not have been strongly motivated to be accurate, as there were no incentives for correct predictions or accurate judgments of others’ predictions. Second, it is unclear how challenging the knowledge questionnaires were, and so gauging participants’ expertise is difficult. It seems clear that objectively assessed experts within each sample were more knowledgeable than the rest of those samples, and that participants in Study 3 were more knowledgeable about basketball than were the populations from which the participants in Studies 1 and 2 were drawn, but absolute levels of knowledge are unknown. Third, we deviated from our preregistered plan in Study 3 to respond to recruitment challenges and because of the sample’s expertise. Study 3, then, should be considered somewhat exploratory.

## Conclusion

We tested whether the “surprisingly popular” method could crowdsource accurate predictions of future outcomes. Applied to non-expert samples (or self-assessed expert subsamples), the method did not yield better predictions than other aggregative approaches. However, when applied to the predictions made by objectively assessed experts, the SP method was consistently (if narrowly) the most effective method. Although there is more to learn about the conditions under which SP is most effective, it shows promise as a means of crowdsourcing predictions of future outcomes.

## Supplementary information

**Additional file 1:** Inferential tests, analyses using forecasting accuracy as a proxy for expertise, notes on overlap between expert criteria, and examinations of expert subsamples.

## Data Availability

The datasets generated and/or analyzed during the current studies are available in each study’s Open Science Framework (OSF) repository (Study 1, https://osf.io/y7z6s/; Study 2, https://osf.io/x8s76/; Study 3, https://osf.io/2rzcs/).

## Abbreviation

SP: Surprisingly popular

## Footnotes

1 The average metacognitive judgments are weighted by the proportion of participants who gave each answer, thusly: (.30 * .15 + .70 * (1 − .90) * 100) = 11.5.

2 (.30 * 4.5) = 1.35 versus (.70 *3.5) = 2.45.

3 In a deviation from the preregistration, we began a week later than anticipated due to survey programming delays.
