# Supplementary material for: Does the “surprisingly popular” method yield accurate crowdsourced predictions?
Source: Cogn Res Princ Implic. 2020 Nov 11;5:57. doi: 10.1186/s41235-020-00256-z (PMC7658271; doi:10.1186/s41235-020-00256-z)
Supplement: Supplementary file 1 — Additional file 1: Inferential tests, analyses using forecasting accuracy as a proxy for expertise, notes on overlap between expert criteria, and examinations of expert subsamples. [file 41235_2020_256_MOESM1_ESM.docx]

Supplemental Material

Study 1: Inferential tests, analyses using forecasting accuracy as a proxy for expertise,

and notes on overlap between expert criteria

The preregistration for Study 2 specified that we would conduct binomial sign tests to compare aggregation methods to one another (consistent with the approach of Prelec et al., 2017, but inconsistent with the approach of Lee et al., 2018). We explicitly did not preregister this approach for Study 1, as these inferential tests are quite low in sensitivity. (Specifically, the post hoc power of the tests comparing the democratic method to the confidence-weighted method was .42 in the whole sample and .48 among objective experts; post hoc power comparing the democratic method to the surprisingly popular method was .56 in the whole sample and .11 among objective experts.) However, we present them here for completeness. The test comparing methods, the McNemar test, requires two outcomes (correct or incorrect prediction); following the approach in Lee et al. (2018, fig. 5), we consider cases in which the method did not make a prediction (i.e., ties) to be incorrect predictions for the purposes of these analyses.

In the total sample, neither the confidence-weighted (*p =* .29) nor surprisingly popular (*p =* .18) methods were significantly different from the democratic method. Among objectively-assessed experts, neither the confidence-weighted (*p =* .37) nor surprisingly popular (*p =* .54) methods were significantly different from the democratic method.

In Study 1, the 80^th^ percentile of accuracy was 57.1%. Participants with performance above this level were considered “demonstrated experts”. This criterion was not preregistered, and, because it depends on a retrospective calculation, cannot be used in practice to predict future outcomes; nevertheless, we include it here and aggregate these participants’ estimates in Table S1.

We also include an alternative measure of accuracy, the kappa coefficient, in Tables S1-S3. (We do not include kappa for self-assessed experts in Study 1, for which N=6, nor any subsamples in Study 3, for which N < 33.)

Table S1: Performance (correct predictions, % correct) across aggregation methods with additional expertise criterion.

|  | Individual | Democratic | Confidence-weighted | Surprisingly Popular |  |
| --- | --- | --- | --- | --- | --- |
| Total sample | 52.8% | 116.5, 53.9%, kappa = .08 (SE = .07) | 120, 55.6%,  kappa = .11 (SE = .07) | 111, 51.4%,  kappa = .03  (SE = .07) |  |
| Self-assessed experts | 58.0% | 127, 58.8% | 125.5, 58.1% | 120, 55.6% |  |
| Objectively-assessed experts | 54.9% | 127.5, 59%,  kappa = .18  (SE = .07) | 129, 59.7%,  kappa = .19  (SE = .07) | 130, 60.2%,  kappa = .20  (SE = .07) |  |
| Demonstrated experts | 57.1% | 144, 66.7%,  kappa = .33  (SE = .06) | 144, 66.7%,  kappa = .33  (SE = .06) | 144, 66.7%,  kappa = .33  (SE = .06) |  |
| NFLpickwatch.com | 62.3% | 140.5, 65.0%,  kappa = .30  (SE = .06) |  | | |
| Fivethirtyeight.com | 134, 62.0%,  kappa = .24 (SE = .07) | | | | |

In addition, we examined the degree of overlap between different methods of assessing expertise. Six participants (all of the self-assessed experts) were both self-assessed and objectively assessed experts; the two measures were correlated at .66. Four participants were both self-assessed and demonstrated experts; self-assessed expertise and performance were correlated at .20. Fifteen participants were both objectively assessed and demonstrated experts; objectively assessed expertise and performance were correlated at .21.

Study 2: Inferential tests, analyses using forecasting accuracy as a proxy for expertise,

and notes on overlap between expert criteria

As noted, for Study 2, we preregistered that we would conduct two-tailed binomial sign tests (McNemar’s test) to compare methods to one another. We no longer believe that these tests are particularly informative because of their low sensitivity (post hoc power for the relevant tests ranged from .10 to .67), but we present them here. In the total sample, neither the confidence-weighted (*p =* 1.00) nor surprisingly popular (*p =* 1.00) methods were significantly different from the democratic method. Among self-assessed experts, neither the confidence-weighted (*p =* .72) nor surprisingly popular (*p =* .22) methods were significantly different from the democratic method. Among objectively-assessed experts, neither the confidence-weighted (*p =* 1.00) nor surprisingly popular (*p =* .22) methods were significantly different from the democratic method.

In Study 2, the 80^th^ percentile of accuracy was 72%. Participants with performance at or above this level were considered “demonstrated experts,” and their estimates are aggregated in Table S2.

Table S2: Performance (correct predictions, % correct) across aggregation methods with additional expertise criterion.

|  | Individual | Democratic | Confidence-weighted | Surprisingly Popular |
| --- | --- | --- | --- | --- |
| Total sample | 60.8% | 87, 79.1%,  kappa = .58 (SE = .08) | 87.5, 79.6%,  kappa = .59 (SE = .08) | 88, 80.0%,  kappa = .60  (SE = .08) |
| Self-assessed experts | 68.2% | 85, 77.3%,  kappa = .55  (SE = .08) | 84, 76.4%,  kappa = .53  (SE = .08) | 79, 71.8%,  kappa = .44  (SE = .09) |
| Objectively-assessed experts | 67.0% | 87.5, 79.6%,  kappa = .59  (SE = .08) | 88, 80.0%,  kappa = .60  (SE = .08) | 91, 82.7%,  kappa = .65  (SE = .07) |
| Demonstrated experts | 77.6% | 95, 86.4%,  kappa = .73  (SE = .07) | 93, 84.5%,  kappa = .69  (SE = .07) | 95, 86.4%,  kappa = .73  (SE = .07) |
| Fivethirtyeight.com | 95, 86.4%,  kappa = .73 (SE = .07) | | | |

In addition, we examined the degree of overlap between different methods of assessing expertise. Nineteen participants were both self-assessed and objectively assessed experts; the two measures were correlated at .44.

Twelve participants were both self-assessed and demonstrated experts; self-assessed expertise and performance were correlated at .16. Fifty-two participants were both objectively assessed and demonstrated experts; objectively assessed expertise and performance were correlated at .42.

Study 3: Inferential tests and examinations of expert subsamples

*Total sample*

We computed McNemar’s test to compare the surprisingly popular method to the democratic method; the methods were significantly different (*p* = .006). The confidence-weighted method, however, was not significantly different from the democratic method (*p* = .23). As noted in the main text of the manuscript, the total sample in Study 3 was already quite expert. Moreover, the subsample analyses are based on very small samples, sometimes from as few as 2 participants. Thus, we present these subsample analyses for consistency with our preregistration plan, but do not believe that they are informative.

*Self-assessed experts*

Participants who rated their knowledge of NBA basketball as “extremely knowledgeable” on a 5-point scale were considered *self-assessed experts*; their predictions were analyzed and aggregated separately. Twenty-one of the 130 participants, or 16.2% of the sample, met this criterion. As the self-evaluation was done at the outset of the study and participants could choose whether to make predictions each week, the total number and proportion of self-assessed experts varied from week to week. As noted in the main text, quite often only one self-assessed expert made a prediction in a given week, meaning that “aggregations” in these weeks was not possible.

Consistent with Studies 1 and 2 (but in contrast to the findings of Lee et al., 2018), aggregations of the judgments of the subsample of self-assessed experts generally did not outperform the sample as a whole. As shown in Table 4, the democratic method and SP method produced slightly worse predictions than the overall sample; the confidence-weighted method produced slightly better predictions than the overall sample. Comparing across methods, the confidence-weighted method performed best. Among self-assessed experts, the confidence-weighted method was significantly different from the democratic method (*p =* .04), but the surprisingly popular method was not (*p =* .44).

*Objectively-assessed experts*

Participants scoring in the top quintile of the knowledge quiz were considered objectively-assessed experts. The 80^th^ percentile of quiz scores was 15/19; this yielded 42 objectively-assessed expert participants. As was the case for self-assessed experts, because participants could choose whether to make predictions each week, the total number and proportion of objectively-assessed experts varied each week.

As shown in Table S3, aggregating the judgments of objectively-assessed experts produced predictions that were more accurate than the overall sample when using the democratic and confidence-weighted methods (2 games, 0.4%). However, using the SP method led to predictions that were worse than the overall sample (7.5 games, or 2.0%). Among objectively-assessed experts, the confidence-weighted method was significantly different from the democratic method (*p =* .004), but the surprisingly popular method was not (*p =* .58); note that, as described above, the McNemar test disadvantages the democratic method (which yields more ties) by counting ties as incorrect predictions.

*Demonstrated experts*

In Study 3, the 80^th^ percentile of accuracy was 66.8%. 23 participants had accuracies exceeding this criterion, and were thus classified as demonstrated experts. From 1 to 11 demonstrated experts made predictions each week. Across all prediction methods, the demonstrated experts outperformed the total sample, self-assessed experts, and objectively-assessed experts. When examining accuracy, neither the confidence-weighted nor the surprisingly popular method appreciably outperformed the simple democratic method in aggregating these predictions; however, using McNemar’s test (which counts ties as incorrect), the surprisingly popular method outperformed the democratic method (*p* = .004) though the confidence-weighted did not (*p* = .27). The complete results can be found in Table S3.

Table S3: Performance (total correct predictions, % correct) across aggregation methods.

|  | Individual | Democratic | Confidence-weighted | Surprisingly Popular |
| --- | --- | --- | --- | --- |
| Total sample | 61.8% | 306.5, 65.9%,  kappa = .32 | 304.5, 65.5%,  kappa = .31 | 313, 67.3%,  kappa = .35 |
| Self-assessed experts | 63.0% | 304, 65.4% | 307.5, 66.1% | 299, 64.3% |
| Objectively-assessed experts | 63.5% | 311.5, 67.0% | 313.5, 67.4% | 304, 65.4% |
| Demonstrated experts | 69.0% | 315.5, 67.8%, | 316.5, 68.0%, | 315, 67.7%, |
| Fivethirtyeight.com | 317, 68.2%,  kappa = .36 | | | |
